# The effectiveness and safety of combining varenicline with nicotine e-cigarettes for smoking cessation in people with mental illnesses and addictions: study protocol for a randomised-controlled trial

**DOI:** 10.1186/s12889-018-5351-7

**Published:** 2018-05-04

**Authors:** Chris Bullen, Marjolein Verbiest, Susanna Galea-Singer, Tomasz Kurdziel, George Laking, David Newcombe, Varsha Parag, Natalie Walker

**Affiliations:** 1 National Institute for Health Innovation, School of Population Health, University of Auckland, Private Bag 92019, Auckland, 1142 New Zealand; 2 Centre for Addiction Research, School of Population Health, University of Auckland, Private Bag 92019, Auckland, 1142 New Zealand; 3 Community Alcohol & Drug Services, Waitemata District Health Board, Pitman House, 50 Carrington Road, Point Chevalier, Auckland, 1003 New Zealand; 4 School of Medical Sciences, Faculty of Medical and Health Sciences, The University of Auckland, Private Bag 92019, Auckland, 1142 New Zealand; 5 Department of Social and Community Health, School of Population Health, University of Auckland, Private Bag 92019, Auckland, 1142 New Zealand

**Keywords:** Varenicline, Electronic cigarettes, E-cigarettes, Smoking cessation, Effectiveness, Safety, Randomised trial, Mental illness, Addiction

## Abstract

**Background:**

Smoking rates are higher in New Zealand (NZ) adults with mental illnesses and alcohol and other drug (AOD) addictions, compared to the overall population. Quit attempts using “gold standard” smoking cessation treatments often fail in people with these conditions, so more flexible treatment regimens that adapt to a person’s responsiveness to treatment are worth investigating. The STATUS trial aims to evaluate the effectiveness and safety of combining varenicline with nicotine e-cigarettes for smoking cessation among varenicline non-responders in treatment for mental health illnesses and/or AOD addictions.

**Methods:**

This is a pragmatic two-arm, open-label, randomised trial. Participants will be daily smokers using mental health and/or addiction services in Auckland, aged ≥18 years, motivated to quit smoking, and eligible to access varenicline through the NZ special authority process. After 2 weeks of using varenicline plus behavioural support, participants who have not reduced their daily smoking by ≥50% will be randomised (1:1) to either 10 weeks of continued varenicline use or 10 weeks of varenicline plus an 18 mg/mL nicotine e-cigarette. All participants will receive weekly withdrawal-orientated behavioural support calls for 6 weeks post-randomisation. The primary outcome is self-reported biochemically-verified (exhaled carbon monoxide) continuous abstinence at 24 weeks post-randomisation. Secondary outcomes, measured at six, 12 and 24 weeks post-randomisation include: self-reported continuous abstinence, 7-day point prevalence abstinence, smoking reduction, time to relapse, cross-over, use of other smoking cessation support, serious adverse events, treatment adherence, compliance, acceptability, dual use, continuation of treatment use, mental illness symptoms and AOD use, health-related quality of life, and cost-analysis. A sample size of 338 will confer 80% power (*p* = 0.05) to detect a 15% absolute difference between the varenicline alone and varenicline plus e-cigarette groups.

**Discussion:**

People with mental illness and/or AOD addictions are just as motivated as others to quit smoking, but are less likely to succeed. Adapting smoking cessation medication after a lack of responsiveness in the first 2 weeks of initial treatment in this priority population by adding a nicotine e-cigarette may be one way to increase long-term quit rates.

**Trial Registration:**

Australian NZ Clinical Trial Registry: ACTRN12616001355460 (29 September 2016).

**Electronic supplementary material:**

The online version of this article (10.1186/s12889-018-5351-7) contains supplementary material, which is available to authorized users.

## Background

Smoking prevalence in the New Zealand (NZ) adult population has declined from 25% in 1996 to 16% in 2017 [1], but among people with mental illnesses and/or alcohol and other drug (AOD) addictions the prevalence of smoking has remained far higher: among people with a self-reported mental illness there was only a slight decrease from 33% in 1996, to 29% in 2012 [2]. Conversely, the proportion of smokers in NZ with a mental illness has increased from 27% in 1996, to 47% in 2012 [2]. More recently, disproportionately higher rates of tobacco use were reported among people with mental illnesses including schizophrenia (60–85%), bipolar disorder (51–70%), major depression (36–80%), and anxiety disorders (32–60%), alcohol disorders (35–80%) and other addictions (49–98%) [3]. In 2012, approximately 27% of NZ smokers reported having an AOD addiction [2], and more than half of people with a substance use disorder were current smokers [3, 4]. Worldwide, an estimated 84% of people receiving AOD treatment smoke tobacco [5].

Although smokers with mental illnesses and/or AOD addictions are just as motivated as others to quit smoking [6] they are less likely to succeed [4, 7, 8]. Reasons include a higher level of nicotine dependence and a greater likelihood of experiencing additional factors known to impede quitting, such as lower socioeconomic status and comorbidities [8]. In NZ, smoking cessation support for smokers with these problems is the same as offered to other smokers, namely brief advice to stop smoking, referral for behavioural support (e.g. Quitline) and a prescription for nicotine replacement treatment (NRT), bupropion (Zyban®), or nortriptyline (Norpress®). Smokers who have previously failed to quit using these medications may then be prescribed varenicline (Champix® - the most effective, but most costly smoking cessation medication currently available) [9]. However, 85% of quit attempts by smokers using these ‘gold standard’ treatments ultimately fail [10], in part due to the predominant ‘one-size-fits-all’ treatment model for smoking cessation which overlooks the considerable between-person heterogeneity in response to treatment [11, 12]. Smokers with a history of mental illness and/or AOD addiction may require a much more individualized treatment approach.

Unfortunately, the vast majority of clinical trials examining the effects of pharmacotherapy on smoking cessation have excluded smokers with a history of mental illness and/or AOD addictions, and the few trials that have focused on this population have typically been uncontrolled or underpowered [8]. Data from these trials suggest that varenicline can be recommended for smokers with current or a history of co-existing problems [8, 13–17]. Varenicline significantly increases continuous abstinence rates compared to placebo in smokers with depression (one randomised controlled trial [RCT]; *N* = 525; odds ratio [OR] 2.53; 95% confidence interval [CI] 1.56–4.10) [18] and bipolar disorder (one RCT; *N* = 60; OR 8.1; 95% CI, 2.03–32.5) [19], but not in smokers with schizophrenia (three RCTs, *N* = 322, RR = 0.79, 95% CI 0.58–1.08, *p* = 0.14) [20]. There is now ample evidence from a range of sources indicating that varenicline is not associated with mental illness-related adverse events or worsening of symptoms. The recently published EAGLES trial (*N* = 8144) found no significant difference in neuropsychiatric events between participants randomised to varenicline, bupropion or NRT compared to placebo, including in those with a history of psychiatric illness [14].

A meta-analysis pooling the findings of 12 trials showed an increase in smoking abstinence with the use of combination pharmacotherapy compared to monotherapy (12 RCTs, *N* = 5183, OR 1.44, 95%, CI 1.15–1.86) [21]. Combination therapy involving varenicline and NRT may increase abstinence rates compared to monotherapy [22]. One trial (*N* = 117) showed that combining a nicotine patch with varenicline improved quit rates (with no observed increase in adverse events) [23]. Adding a nicotine patch has also been found to increase the short- to mid-term efficacy of varenicline among smokers without co-existing conditions [24]. The rationale for this combination is that varenicline targets primarily the alpha4-beta2 and alpha7 nicotinic receptors; therefore, adding nicotine would bind to the remaining nicotine receptors, aiding the de-conditioning of cigarette smoking and reinforcement.

There is, however, limited evidence on the effectiveness of combining varenicline with nicotine delivered by e-cigarettes (battery powered devices that deliver an aerosol of propylene glycol or glycerine, nicotine, and flavours, hereafter referred to as ECs). One small study (*N* = 69) that evaluated ECs used together with varenicline at a specialist stop smoking clinic for highly dependent smokers found them to be more effective in combination - participants who used varenicline plus ECs were more likely to be abstinent 4 weeks after their target quit date than those who used ECs alone (85% versus 54%, *p* < 0.05) [25] - and detected no adverse events related to their combination. No information is available on the impact of combining varenicline with nicotine ECs on smoking abstinence in people with mental illnesses and/or AOD addictions.

The evidence from EC trials is that safety issues are unlikely to be a significant problem. The ASCEND trial (*N* = 657) found no difference in adverse events at 6 months between EC users and NRT users [26]. Specifically, no mental health related adverse events were detected in previous EC clinical trials [27]. Some health risks may emerge from long-term use of ECs but, given what is known about the constituents of the vapour produced by ECs, it is generally agreed that in the unlikely event of health effects occurring, they would be minor in comparison to continued smoking [28]. ECs are both efficacious for cessation when delivering nicotine [25–29] and highly acceptable to patients who smoke and who are undergoing treatment for other addictions, an important feature because of the typically low uptake of conventional smoking cessation treatment in this population. Secondary analysis of our EC trial found that ECs with nicotine had similar efficacy to (and were preferred over) conventional nicotine patch among smokers with mental illness [30].The Cochrane review of ECs for smoking cessation reported an effect only in ECs with nicotine, similar to that of NRT [27].

In NZ nicotine is regulated as a medicine, except when delivered in tobacco smoke. Currently it is illegal to sell an EC that contains nicotine or to make a cessation claim about ECs, because Medsafe (the authority for licensing medicines) consider ECs as medicines when they are supplied for use as an aid to smoking cessation or when supplied with nicotine (even if they are not represented as aids to smoking cessation). Furthermore, they are not therapeutic products when they are supplied as a “gadget” which consumers may choose to use as a social prop or as an item which is to be used interchangeably with cigarettes. However, in late March 2017, the Ministry of Health announced their plans to legalise the sale and supply of EC and e-liquid to happen from the middle of 2018 [31].

The STATUS trial involves ‘boosting’ treatment in people with mental illness and/or AOD addictions if no response is seen to the initial treatment within 2 weeks. The rationale for this design is based on the finding that smokers who reduce their cigarette consumption by at least 50% during the first 2 weeks after starting on either varenicline or NRT (hereafter, ‘responders’) are 2 to 3 times more likely to quit for the long-term than smokers who do not experience this positive medication response (‘non-responders’) [23, 32–34]. This increased chance of quitting is thought to result from more the rapid de-conditioning of nicotinic-receptor-mediated dopamine reinforcement from cigarette smoke among responders; NRT is a full agonist at most nicotinic receptors and varenicline a partial agonist at the receptor thought to be most important for cigarette reinforcement. It is therefore possible that responders experience a more pronounced and rapid blunting of cigarette reinforcement due to NRT and varenicline nicotinic receptor occupancy than do non-responders, which ultimately lessens the reinforcing potential of cigarettes. Furthermore, only about a third of patients who use varenicline or NRT are positive responders, suggesting the majority of smokers trying to quit receive sub-optimal treatment [33].

The logical next step then is to attempt to ‘restore’ quit rates among non-responders to that of responders. Two RCTs (total *N* = 557) showed that NRT non-responders (without co-existing disorders) were randomised to receive either an alternative (varenicline) had significantly higher six-month quit rates than NRT responders who remained on NRT alone (varenicline - OR 2.80; 95% CI, 1.11 to 7.06) [33, 34]. Such ‘adaptive’ treatment regimens have generated positive outcomes for medications involved in managing schizophrenia [35], depression [36], and alcohol dependence [37], but to date there are no trials using an adaptive treatment for smoking cessation in people with mental illness and/or AOD addictions. The STATUS trial investigates the effect of combining varenicline with nicotine-containing ECs over varenicline alone. We hypothesize that combining varenicline with nicotine ECs will result in higher smoking abstinence rates in varenicline non-responders with mental illnesses and/or AOD addictions, compared to those that continue using varenicline only.

### Changes to the original study protocol

Initially we designed the trial to include a third arm of participants randomised to varenicline plus bupropion. There is some evidence that combination treatment with bupropion is superior to monotherapy [38]. However, while this design was supported by the ethics committee it was not subsequently supported by clinical groups in health services in the region, because of concerns about adverse effects in a highly medicated population group among whom there is also a high prevalence of medical conditions that might present additional risks (such as epilepsy) should a participant be randomised to the varenicline plus bupropion arm. Without their support we were unlikely to meet recruitment targets, so elected to remove this arm from the study.

## Methods

### Design

**Fig. 1:**
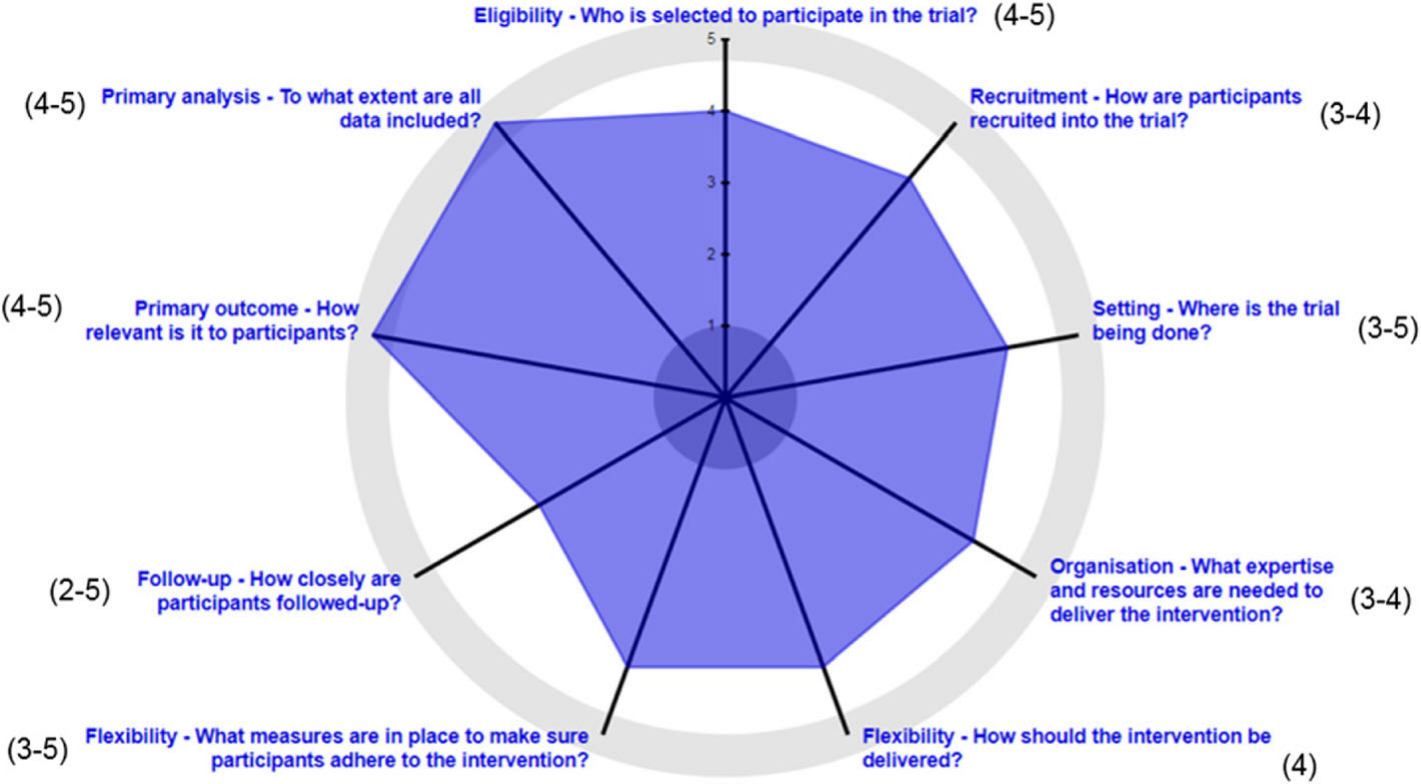
PRECIS-2 wheel showing the pragmatic character of the STATUS Trial

The following trial protocol (version 4.0, 1 November 2017) adheres to the SPIRIT guidelines (Additional file 1). STATUS is an open-label, two-arm, pragmatic, community-based, randomised controlled trial. The pragmatic character of the trial is depicted in Fig. 1, showing the nine domains of the PRECIS-2 wheel (PRagmatic-Explanatory Continuum Indicator Summary-2), namely: 1) eligibility, 2) recruitment, 3) setting, 4) organization, 5) flexibility (delivery), 6) flexibility (adherence), 7) follow-up, 8) primary outcome, and 9) primary analysis [39]. Each domain was scored independently by five authors (CB, MV, VP, GL, DN) on a 5-point Likert scale ranging from 1 “very explanatory” to 5 “very pragmatic”. The average scores are shown in Fig. 1 (ranges between brackets).

### Study population

Daily smokers who are motivated to quit, are outpatients currently receiving treatment for mental illness and/or AOD addictions from community alcohol and drug services (CADS), community mental health services (CHMS) and/or non-governmental organisations (NGOs) in the Auckland region of NZ, and meet the additional eligibility criteria below.

### Eligibility criteria

Participants will be eligible if they have access to a telephone, and are: ≥18 years, able to provide written consent, and prepared to use varenicline alone or with a nicotine EC. Participants also need to be eligible for fully subsidised varenicline under the NZ Ministry of Health’s special authority process (i.e., they have to have tried to quit at least twice before with NRT, and have not used varenicline in the past 12 months [40]). Only one person per household is eligible. Pregnant women and women who are breastfeeding will be excluded from the trial, as will current users of other nicotine-based and non-nicotine based cessation therapies, including NRT, ECs (with or without nicotine), bupropion, clonidine, nortriptyline or varenicline. People will also be excluded from the trial if they have any contraindications for the use of varenicline (i.e. a heart attack, stroke or severe angina within the previous 2 weeks) or ECs (i.e. a self-reported history of severe allergies and/or poorly controlled asthma).

### Recruitment

Participants will be recruited from CADS and CMHS clinics as well as from NGOs that provide community mental health and addiction services in the Auckland metropolitan region of NZ (total population 1.377 million). Potential participants will be invited to consider taking part in the trial using posters and fliers in clinics. People interested in the trial will be directed to contact the study centre at the University of Auckland’s National Institute for Health Innovation (NIHI) by telephone, email, text, Facebook or through the study website. Clinicians will also actively inform their clients about the trial during routine consultation. As is current best practice, clients who smoke will be advised to quit smoking and to consider taking part in the trial as one possible route to do so. If the client is interested in the study and would like to know more, the service provider will ask the client for their verbal consent to provide their name and phone number to the research team. Potential participants will also be identified via searching the client database within the clinics. Once identified, potential participants will be invited by letter and followed up by phone (either by someone from the clinical team or by a researcher, calling on behalf of the clinic).

### Randomisation, allocation concealment and sequence generation

Participants who fulfil entry criteria and who have not responded to 2 weeks of varenicline as detailed below will be randomly allocated in a 1:1 ratio by a central computer to one of the two study groups, using block randomisation with varying block sizes. The randomisation sequence will be prepared by the study statistician and loaded into a secure database.

### Blinding

This is an open-label trial. The obvious physical differences between treatment arms prevent blinding of both participants and research staff.

### Study interventions

People that are interested in taking part in the study will be screened for eligibility over the phone by a research assistant. After screening, a study doctor will perform a review of the medical information collected during the screening to confirm the participant’s eligibility. Eligible participants will attend a face-to-face meeting with a research assistant (to build trust and rapport), where baseline data will be collected, informed consent will be obtained, and 10–15 min of withdrawal-orientated behavioural support will be delivered. For convenience, the meeting will be onsite at the participant’s usual clinic. A prescription for a 12-week course of varenicline (a 2 week supply of varenicline tablets 0.5 mg and 1 mg to up-titrate, plus a 2 week supply of 1.0 mg tablets to continue thereafter) will be faxed to the participant’s pharmacy of choice. Participants will be advised to set a quit date in the first week after they have started their varenicline treatment, continue to smoke up to this quit date (if they wish to), and aim to quit all smoking by the end of the 2 weeks period. The research assistant will phone participant’s 1 week after they have started their treatment, in order to deliver 10–15 min of withdrawal-orientated behavioural support (Table 1).

**Table 1:**
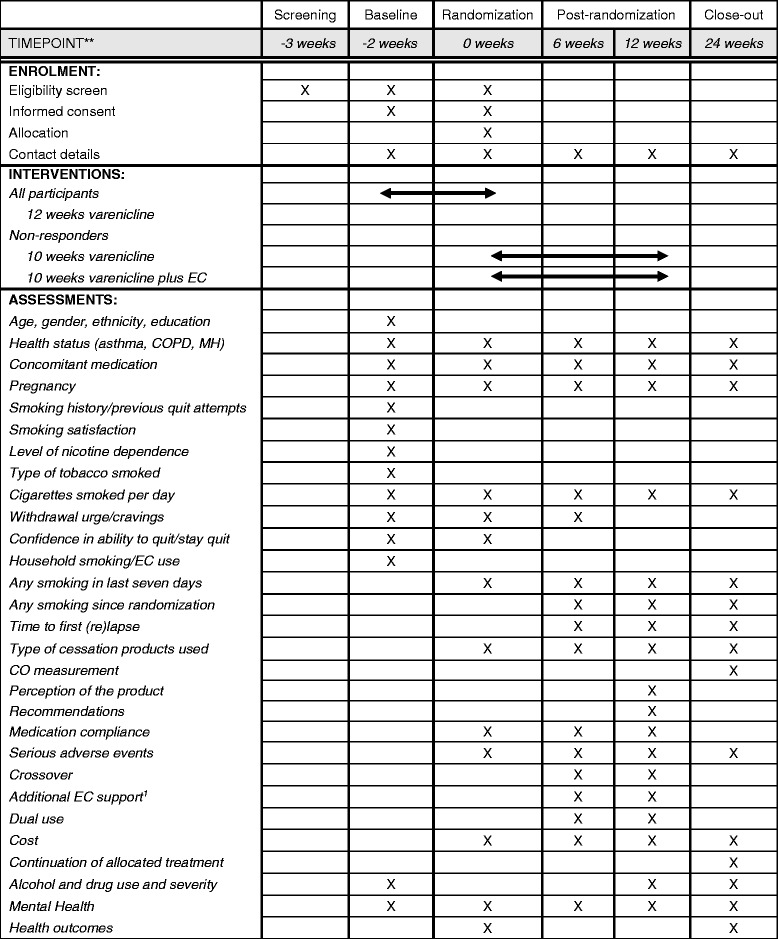
Schedule of enrolment, interventions, and assessments

*COPD* Chronic Obstructive Pulmonary Disease, *MH* Mental Health, *EC* E-cigarette

^1^Only in those allocated to the varenicline + EC group

The research assistant will phone participants two weeks after they have started their treatment, to assess their response to varenicline. Those who are varenicline responders (i.e. they have quit smoking or reduced the number of cigarettes smoked per day by > 50%) will be advised to continue their use of varenicline for another 10 weeks and to contact Quitline and/or a health professional (general practitioner or head clinician) for on-going smoking cessation behavioural support. Responders will not progress further in the study but simply continue on with their course of varenicline. Varenicline non-responders (i.e. those who are still smoking and have not reduced the number of cigarettes smoked per day by ≥50%) will be randomised to one of two treatment arms: 10 weeks of continued varenicline use (*n* = 169) or 10 weeks of continued varenicline use plus 18 mg/mL nicotine EC (n = 169) (Table 1).

#### Varenicline only

This ‘usual care’ group will remain on varenicline (one 1.0 mg tablet, twice a day) for another 10 weeks. As part of the NZ special authority process to assess 12 weeks of subsidized varenicline, pharmacies are required to dispense varenicline in three lots over 8 weeks: 1) an initial two-week starter pack plus 2 weeks maintenance treatment, 2) 4 weeks maintenance treatment, and 3) 4 weeks maintenance treatment. Consequently, participants will need to pick up two further batches of varenicline from their pharmacy: one at 2 weeks post-randomisation, and another at 6 weeks post-randomisation.

#### Varenicline plus nicotine EC

This group will remain on varenicline (one 1.0 mg tablet twice a day) for another 10 weeks (as outlined above), but will be couriered a tank-type EC and sufficient e-liquid containing nicotine. The Joyetech AIO D16 starter kit was selected following consultation with community-based smoking cessation providers (who work with smokers with mental illnesses and/or AOD addictions), and with NZ EC retailers about the most suitable EC for the trial population. This device was universally regarded highly because of its reliability, long battery life and popularity with vapers. Participants will be provided with a flavoured e-liquid manufactured in NZ, with a flavour similar in taste to a popular brand of factory-made cigarettes. The nicotine content of the e-liquid used in the trial will be independently assessed to verify nicotine content is as labelled and to check for contaminants, with the aerosol also checked for contaminants that may be generated by heating [29]. Participants will be provided with written instructions on how to assemble and use their EC, plus provided with a web-link to short on-line instruction videos hosted by the NZ based on-line EC retailer. This retailer will also provide a helpline number for participants to call should they need additional help or advice regarding use of the EC. The videos and helpline reflects ‘real world’ support offered by the vaping community in NZ for new users of ECs.

#### Behavioural support

At the time of randomisation, all participants will be advised to set a new quit date. Post-randomisation, all participants will receive six weekly behavioural support calls from a research assistant. During these calls, data on treatment use, smoking behaviour and adverse events will be collected.

### Baseline assessments

The following baseline data will be collected (Table 1):

- *Demographics:* Date of birth, gender, ethnicity, and socio-economic position (based on highest level of education attained);
- *Smoking history*: Age when started, number of (roll-your-own [RYO]) cigarettes smoked per day, number of years as regular smoker, number of previous unsuccessful attempts to give up in past 12 months and the method used, type of cigarettes smoked per day (e.g. roll-your-own or factory-made). A participant who smokes RYO cigarettes may say that their cigarettes vary in size. A RYO-to-cigarette conversion calculator will be put in place to calculate the number of ‘standard’ RYO cigarettes smoked based on the amount of grams of loose tobacco they use [41];
- *Level of cigarette dependence*: Measured by the Fagerström Test for Cigarette Dependence (FTCD) Questionnaire [42, 43];
- *Confidence in ability to quit*: Measured on a Likert scale of 1–5, where 1 = not very and 5 = very;
- *Smoking satisfaction*: Measured using the Modified Cigarette Evaluation Questionnaire (mCEQ) [44];
- *Other smoking related information*: Household smoking and EC use;
- *General health*: Mouth ulcers, shortness of breath, cough, asthma, Chronic Obstructive Pulmonary Disease (COPD), and current or history of mental health (including depression, schizophrenia, and anxiety);
- *AOD use/severity of AOD problems*: Measured using a two-question version of the World Health Organization’s Alcohol, Smoking and Substance Involvement Screening Test (ASSIST-FC) [45];
- *Mental health symptoms*: Measured using the Kessler Psychological Distress Scale (K6) [46];
- *Concomitant medication*: Information about types of medication currently used will be collected;
- *The physical signs and symptoms associated with withdrawal****:*** Measured using the Mood and Physical Symptoms Scale (MPSS) [47], including urge to smoke.

### Primary outcome

The primary outcome is 24 week continuous abstinence (Russell Standard) defined as self-report of smoking not more than five cigarettes from the randomisation date, supported by biochemical validation (eCO reading using a Bedfont Smokerlyser CO Monitor, with a reading of ≤10 ppm signifying abstinence). Sensitivity analysis will be undertaken looking at different cut-offs for the CO measurement, given a lack of consensus about the best cut-off to use [48, 49].

### Secondary outcome measures

The following secondary outcome measures will be assessed at randomisation, then at six, 12 and 24 weeks post-randomisation (Table 1):

- *Continuous abstinence (6 and 12 weeks)*: The proportion of participants that have stopped smoking defined as smoking not more than five cigarettes in total from randomisation (self-reported) (biochemically validated using a CO reading of ≤10 ppm at 24 weeks);
- *Seven-day point prevalence abstinence*: The proportion of participants that have stopped smoking, defined as self-report of smoking no cigarettes at all, not even a single puff in the last seven days;
- *Time to first lapse*: Defined as time to first cigarette smoked, even a single puff;
- *Time to first relapse*: Defined as time to smoking more than five cigarettes a day for three or more days in a row;
- *Change from randomisation in cigarettes smoked per day*: If the participant is still smoking;
- *Smoking reduction*: Defined as reducing consumption by ≥50% (in terms of numbers of cigarettes per day, weight of loose tobacco per day, or when smoking for non-daily smokers);
- *Change from randomisation in the physical signs and symptoms associated with withdrawal (randomisation and 6 weeks)***:** Measured using the Mood and Physical Symptoms Scale (MPSS) [47], including urge to smoke;
- *Concomitant medication*;
- *Use of any other smoking cessation methods*: NRT, non-NRT methods of cessation such clonidine, nortriptyline, acupuncture, Quitline, etc.;
- *Alcohol and drug use/severity of AOD problems (12 and 24 weeks only)*: Measured using the ASSIST-FC [45];
- *Mental health symptoms*: Measured using the K6 [46];
- *Health-related quality of life (randomisation and 24 weeks only):* Measured using the NZ EQ-5D Tariff 2 (telephone script) [50];
- *Confidence in ability to quit (randomisation only):* Measured on a Likert scale of 1 to 5, where 1 = ‘not very’ and 5 = ‘very’;
- *Adverse events*: Information regarding adverse events and whether they are related to treatment;
- *Medication compliance (randomisation, 6 and 12 weeks):* Self-reported frequency of using allocated product;
- *Crossover*: Participants in the varenicline only or varenicline + bupropion groups will be asked whether they accessed and used an EC (with or without nicotine) in the follow-up period;
- *Additional EC support (6 and 12 weeks):* Participants allocated to the varenicline + EC group will be asked whether they accessed any (on-line) EC support networks or bought different e-juice or EC during the trial;
- *Dual use (6 and 12 weeks):* Defined as use of both their allocated treatment and continued smoking of cigarettes;
- *Continuation of use (24 weeks):* Defined as continued use of their allocated treatment after the end of the designed 12 week treatment period;
- *Perception of the product (24 weeks only):* Participants will be asked for their views on use of their allocated treatment as a smoking cessation aid;
- *Acceptability/Recommendations* (*24 weeks only):* Participants will be asked whether they would recommend their allocated treatment to another smoker who wanted to quit.

### Sample size

A sample size of 338 (169 in each arm) anticipates a 30% loss-to-follow-up and will confer 80% power, 2-sided *p* = 0.05 to detect an absolute difference of 15% between the varenicline plus EC group and varenicline alone group in 24 week carbon monoxide (≤10 ppm) verified abstinence rates, with a predicted abstinence rate in the varenicline plus EC group of 30%. The anticipated 30% loss-to-follow-up is more than the 22% noted in the psychiatric cohort in the EAGLES trial [14] but lower than the 39% found in the Ebbert et al. trial [38]. We estimate 30% of participants will collect and use the prescribed varenicline for the first 2 weeks of the study [51], and that most (around 80%) of this group will be varenicline non-responders after 2 weeks of medication use [23]. Thus, 507 people will need to be recruited into the ‘initial treatment phase’ in order to obtain 338 people to be randomised. We anticipate it will take 16 months to recruit 338 participants.

### Data management

Study data will be collected and managed using REDCap (Research Electronic Data Capture) [52]. The study will be monitored early on during the study (after ten participants have been randomised), at study close-out and twice during the course of the trial.

### Statistical analysis

All statistical analyses will be performed using SAS version 9.4 (SAS Institute Inc. Cary NC), and R [53]. Analyses will be undertaken on an intention-to-treat basis. For the main analyses, all participants lost to follow-up will be presumed to be smoking. Per-protocol analysis will be undertaken for the primary outcome where participants with major protocol violations (e.g. cross-overs treatments, withdrawals, and loss to follow-up) will be excluded. Sensitivity analyses will be carried out to determine the effect of missing data and if the level of missing data is deemed high (i.e. > 20%) use of multiple imputation will be employed. Participant flow through the trial will be described in a Consolidated Standards of Reporting Trials (CONSORT) diagram. The number of withdrawals will be summarised by type, treatment group and time, with reasons where provided. Incidence rates, risk difference, RR and 95% CI will be calculated for all binary outcomes, and groups will be compared using chi-squared tests and multiple logistic regression analysis where appropriate. Continuous outcomes will be analysed using multiple linear regression modelling or non-parametric analysis. K6 and change in cigarettes smoked per day (in non-abstainers) will be analysed using repeated measures models, and will adjust for baseline value. Time to relapse will be analysed using Kaplan Meier curves, log-rank test, and Cox proportional hazards regression analysis. Secondary analyses will be conducted to determine the impact of varying cut-offs used for CO measurements, and secondary analyses performed with cessation rates corrected for discordance between reported and CO-verified cessation. The consistency of effects for pre-specified subgroups will be assessed using tests for heterogeneity. Subgroups will be based on sex, ethnicity, and type of cigarette.

### Cost-effectiveness

Cost outcomes will include cost per quitter, cost per person reducing their daily cigarette consumption, and incremental cost effectiveness ratio should the intervention prove to be effective. Comparative cost-utility will be estimated using the NZ EQ-5D tariff 2 [50]. We will compare our data with data from NZ cessation service providers and international studies. This modelling will take a health sector perspective. To give a societal perspective on the benefits (especially to low-income smokers) tobacco expenditure savings to individual smokers who quit and cut down will also be calculated using data on the daily amount smoked prior to quitting and the price of the particular products smoked. A range of approaches to assessing the benefit of years per life saved will also be explored.

### Ethical considerations

Ethics approval for the trial was obtained on 26/09/2016 from the Southern Health and Disability Ethics Committees (16/STH/153). Two subsequent amendments to the study protocol were approved on 4/09/2017 and 8/12/2017. Approval from the NZ Standing Committee on Therapeutic Trials (SCOTT) was obtained on 25/10/2016 for the use of ECs with nicotine (16/SCOTT/86).

### Trial governance

Trial governance includes a steering committee (on which all authors sit), and a trial management team who will manage the day-to-day processes of the trial, including data management. A Data Safety Monitoring Committee (DMC) will be established for the trial. Members will have no conflicts of interest. The DMC will draw up their own terms of reference, will have clearly defined stopping rules in the event of safety concerns agreed upon by the Committee, and will be guided by statistical monitoring guidelines consistent with GCP. The study statistician will provide the DMC with reports on safety data.

### Dissemination policy

Results will be disseminated regardless of the magnitude or direction of treatment effect. Dissemination will include trial registration, feedback to trial participants, publication in an international journal, national and international media releases at the time of journal publication, and presentations to relevant local, national and international audiences (including health service funders and providers). In NZ this will include but is not limited to the Ministry of Health, District Health Boards (including CADS and CMHS), Primary Health Organisations, NGOs, health professionals, and Māori and Pacific organisations managing patients with mental illness and/or AOD addictions and/or involved in provision of smoking cessation support. Criteria for authorship of any papers rising from the trial will be taken from the International Committee of Medical Journal Editors.

## Discussion

The STATUS trial will be unique in that it is one of the first trials to evaluate a highly practicable, adaptive treatment approach to smoking cessation among smokers with mental illnesses and/or AOD addictions. Current evidence suggests that combining varenicline with NRT (including ECs with nicotine) increases quit rates over varenicline alone, whilst the efficacy of nicotine ECs may be enhanced when used in addition to varenicline. However, these approaches to treatment have not been integrated or trialed among people with mental illnesses and/or AOD addictions who are varenicline non-responders. The findings will be of wide interest given the universal difficulty in helping smokers with such problems to quit. Such people are often excluded from smoking cessation trials with an efficacy goal. If positive, our findings will provide healthcare providers with a simple yet effective tool to increase smokers’ chances of successful cessation via adapting treatment based on self-reported initial response. In addition, if positive, this approach could lead to significant cost savings via reducing medication waste in non-responders (who would otherwise continue with an ineffective treatment). The trial is currently ongoing; recruitment started on 31/05/2017, with recruitment expected to take 18 months. Trial findings are likely to be available July 2019.

## Additional files

Additional file 1: SPIRIT Checklist. (DOC 143 kb)

Additional file 2: Consent Form. (PDF 69 kb)

## Abbreviations

AE: Adverse event
AOD: Alcohol and other drugs
ASSIST-FC: Alcohol, Smoking and Substance Involvement Screening Test Short Form
CADS: Community alcohol and drug services
CI: Confidence interval
CMHS: Community mental health services
CO: Carbon monoxide
COPD: Chronic obstructive pulmonary disease
EC: E-cigarette
FTCD: Fagerstrom Test for cigarette dependence
ITT: Intention-to-treat
K6: Kessler Psychological Distress Scale
mCEQ: Modified Cigarette Evaluation Questionnaire
MPSS: Mood and Physical Symptoms Scale
NGO: Non-governmental organisation
NRT: Nicotine replacement therapy
NZ: New Zealand
OR: Odds ratio
RCT: Randomised controlled trial
REDCap: Research Electronic Data Capture
RR: Relative risk
RYO: Roll your own
SCOTT: Standing committee on therapeutic trials
STATUS: Study of adaptive treatment for users of mental health and addiction services
