# Supplementary material for: The effectiveness and safety of combining varenicline with nicotine e-cigarettes for smoking cessation in people with mental illnesses and addictions: study protocol for a randomised-controlled trial
Source: BMC Public Health. 2018 May 4;18:596. doi: 10.1186/s12889-018-5351-7 (PMC5935940; doi:10.1186/s12889-018-5351-7)
Supplement: Supplementary file 2 — Consent Form. (PDF 69 kb) [file 12889_2018_5351_MOESM2_ESM.pdf]

National Institute for Health Innovation
School of Population Health
The University of Auckland

Tamaki Campus
Morrin Road, Glen Innes, Auckland
Private Bag 92019
Auckland
NEW ZEALAND
Telephone: 09 923 4730
Email: c.bullen@auckland.ac.nz
www.auckland.ac.nz

STATUS Trial: Study of Adaptive Treatment in AOD and MH services Users who Smoke)

Consent Form

Please read the following and sign if you agree

I have read, or have had read to me, and I understand, the Participant Information Sheet.

I have been given sufficient time to consider whether or not to participate in this study.

I have had the opportunity to use a legal representative, whanau/ family support or a friend to help me ask questions and understand the study.

I am satisfied with the answers I have been given regarding the study and I have a copy of this consent form and participant information sheet.

I understand that taking part in this study is voluntary (my choice) and that I may withdraw from the study at any time without this affecting my medical care.

I consent to the research staff collecting and processing my information, including information about my health.

I consent to the research staff contacting my usual doctor / GP about my study medications, if I have side effects, if further information is required for the trial and if it is agreed to be the best way to keep them informed.

I agree to an approved auditor appointed by the New Zealand Health and Disability Ethic Committees, or The University of Auckland or their approved representative reviewing my relevant medical records for the sole purpose of checking the accuracy of the information recorded for the study. I understand that this information will remain confidential.

I understand that my participation in this study is confidential and that no material, which could identify me personally, will be used in any reports on this study.

I understand the compensation provisions in case of injury during the study. These are that, in the unlikely event of being injured while taking part in this study, I may be eligible for compensation from ACC just as I would be if I was injured in an accident at work or at home. I would have to lodge a claim with ACC. If my claim was accepted, I would receive funding to assist in my recovery.
I know who to contact if I have any questions about the study in general.
I understand my responsibilities as a study participant.

**Please tick to indicate your agreement with the above statements.** (If no selected then ineligible for the trial)	Yes ☐	No ☐
**I wish to receive a summary of the results from the trial.**	Yes ☐	No ☐
**If yes I wish to have the summary of results sent to this email/physical address:**____		
I agree to be contacted about future studies for which I may be eligible	Yes ☐	No ☐

Declaration by participant:
I hereby consent to take part in this study.

Participant's name: ______________________________

Signature: ______________________ Date: ______________________

Declaration by member of research team:

I have given an explanation of the research project to the participant, and have answered the participant's questions about it.

I believe that the participant understands the study and has given informed consent to participate.

Researcher's name: ______________________________

Signature: ______________________ Date: ______________________

This study was approved by the Southern Health and Disability Ethics Committee on the 07 November 2016 for three years, reference number 16/STH/153
